# Investigation on hybrid optimization approach for minimizing surface roughness in the machining of Mg/TiC metal matrix composites using spark EDM

**DOI:** 10.1038/s41598-025-18560-3

**Published:** 2025-09-29

**Authors:** Dharmeswar Dash, Devarasiddappa Devarajaiah, Santosh Kumar Dash, Sutanu Samanta, Ram Naresh Rai, Debabrata Barik, Prabhu Paramasivam, Abinet Gosaye Ayanie

**Affiliations:** 1 Department of Mechanical Engineering, GKCIET, Malda, West Bengal 732141 India; 2 Department of Automobile Engineering, RGGP, Itanagar, Arunachal Pradesh 791113 India; 3 Department of Production Engineering, NIT, Agartala, Tripura 799046 India; 4 Department of Mechanical Engineering, Karpagam Academy of Higher Education, Coimbatore, 641021 India; 5 Centre for Energy and Environment, Karpagam Academy of Higher Education, Coimbatore, 641021 India; 6 Department of Research and Innovation, Saveetha School of Engineering, SIMATS, Chennai, Tamil Nadu 602105 India; 7 Department of Mechanical Engineering, Adama Science and Technology University, 2552 Adama, Ethiopia; 8 Department of Mechanical Engineering, NERIST, Nirjuli, Arunachal Pradesh -791109, India

**Keywords:** Mg/TiC composites, EDM, SEM, SR, ANN, JAYA, Engineering, Mechanical engineering

## Abstract

Electrical discharge machining (EDM) is a non-traditional machining technique in which material is extracted as debris from the workpiece due to a spark produced at the interface of the workpiece and electrode (tool). This paper presents the machining of Mg-TiC composites in a servo-controlled spark EDM using a copper tool as electrode material by varying the input process parameters. Surface roughness (SR) was modeled in terms of four input process variables viz. Mg/TiC composites (*WP*), pulse on time (*T*_*on*_), pulse off time (*T*_*off*_) and input current (*I*) using artificial neural network (ANN). The predictive performance of the ANN model was better with 93.05% model accuracy. A hybrid optimization methodology integrating ANN with the Jaya algorithm is proposed for the optimization of EDM process parameters to obtain minimum SR. The proposed optimization methodology obtained an optimum value of SR 2.89 µm at optimal process parameters $WP=8.05\text{\%}$, ${T}_{on}=21.00\mu s$*, *${T}_{off}=75.00\mu s$*,* $I=8.00A$. The performance of the ANN-Jaya integrated optimization methodology was found highly accurate and consistent during the consistency test with lower standard deviation. The increase in pulse on time and input current turns in a rougher surface as observed from surface roughness tester. A study on the surface morphology of the machined component is also presented using scanning electron microscope (SEM) images.

## Introduction

Metal matrix composites (MMCs) are widely used due to their unique properties such as strength, hardness, weight, stiffness, conductivity, corrosion resistance, temperature resistance, etc. MMCs are obtained by the combination of high-strength fiber materials (TiC, Al_2_O_3_, SiC, B_4_C, WC, etc.) with matrix materials (Magnesium, Aluminium, Copper, Titanium, Cobalt, Nickel, etc.). The machining of such high-strength and heat-resistant materials into intricate shapes using conventional machining processes is time-consuming and sometimes difficult to machine to the required shape and size. To overcome these difficulties, non-traditional machining (NTM) methods are employed, which also meet the requirements of dimensional accuracy and surface finish. The most widely used NTM technique is EDM because of the controlled erosion of materials from the workpiece material. It uses heat energy in the form of sparks with a frequency of more than thousands of sparks per second to heat and melts the workpiece. The flushing system removes the debris from the working zone. The major advantage of the EDM process is that intricate shapes can be produced more precisely as compared to conventional machining processes.

Somashekhar et al.^1^ enhanced the micro-EDM process by the integration of artificial neural networks (ANN) and genetic algorithms (GA). Their findings indicated that the predicted model closely corresponded with experimental outcomes. Lin et al.^2^ employed response surface methodology (RSM) alongside a central composite design (CCD) to model surface roughness (SR). Key process parameters affecting SR were determined via analysis of variance (ANOVA). Anitha et al.^3^ established a multi-objective optimization framework for the modeling and optimization of the EDM process. Numerous studies have investigated SR prediction in EDM. Markopoulos et al.^4^ developed an ANN model to predict SR with pulse current and pulse duration as input variables, attaining satisfactory predictive accuracy. Prabhu et al.^5^ incorporated fuzzy logic with CCD to enhance SR in EDM. Likewise, Payal et al.^6^ utilized artificial neural networks to estimate process parameters in die-sinking electrical discharge machining of Inconel 825 for the prediction of surface roughness. Roya et al.^7^ performed an experimental investigation on hybrid metal matrix composites (MMCs) utilizing an Al-6061 matrix reinforced with SiC (10 wt%) and TiB2 (2.5 wt%). They utilized a CCD experimental methodology employing both pure dielectric and powder-mixed dielectric as flushing mediums. Their findings demonstrated that powder-mixed dielectric substantially affected the process, decreasing SR as powder concentration rose across the pulse current spectrum. Muthuramalingam et al.^8^ discovered that a powder-mixed dielectric medium produced a thinner, consistently distributed white coating with a reduced number of microcracks on the machined surface. Jabbaripour et al.^9^ examined the EDM machining of Ti-6Al-4 V alloy under diverse settings, demonstrating that pulse current and pulse-on duration significantly influenced surface integrity. Their research emphasized that pulse energy variations substantially caused surface imperfections, including micro-holes, pits, and cracks. Although enhanced pulse energy augmented the material removal rates, it concurrently resulted in a thicker recast layer and increased microhardness.

Mathematical modeling of *SR* in EDM using regression analysis was attempted by Natarajan et al.^10^ who observed peak current and pulse on time as influential process parameters affecting *SR*. In an experimental study of the EDM process during the machining of Inconel alloy, authors Torres et al.^11^ reported current intensity and pulse duration as dominant process variables affecting *SR*. Chandramouli et al.^12^ created the experimental design using the Taguchi method and utilized ANOVA to examine the impact of the input factors on the response (SR). It was found that the *SR* is inversely proportional to pulse off time but directly proportional to current and pulse on time. Prasanna et al.^13^ carried out an experimental investigation of *SR* on AA7075/SiC MMC at various levels of input parameters. The experimental study showed that surface finish can be improved by decreasing the current. Experimental investigation on MMC (AA7075/SiC) using the EDM process also is reported in the literature by Habib et al.^14^. Sarabjeet et al.^15^ explored the relationship between EDM process parameters and the recast layer as a response variable. The experiments were carried out on three different types of MMCs in different dielectric mediums. The authors obtained a smooth recast layer using a powder-mixed dielectric medium. The application of RSM for modeling and optimization of the EDM process is reported in the literature by Gangil et al.^16^. Heidari et al.^17^ have produced ultra-fine grain (UFG) structured copper tool electrodes by the ECAP method to increase electrical wear resistance and improve the performance parameters in EDM, such as MRR, VEW, and EWR. Lakshmaiya et al.^18^ explored application of EDM with the proper control factors for the machining of functionally graded aluminum matrix composites (FGAMCs). They have reported that following the zone position and pulse on time, the pulse current made the biggest impact on the output performance. The crater generation and erosion mechanism are visible in the SEM micrograph of the EDM-machined surface.

Nguyen et al.^19^ carried out a single-objective optimization based on Taguchi approach on the vibration-assisted DSEDM method for milling SKD61 die steel with a copper electrode. Their findings showed that introducing vibration into the workpiece considerably increased the material removal rate (MRR). Furthermore, low-frequency vibration significantly reduced surface roughness (SR) when compared to the non-vibration condition. Phan et al.^20^ suggested a multi-objective decision-making technique for EDM machining of Ti-6Al-4 V titanium alloy using Taguchi-Data Envelopment Analysis based Ranking (DEAR) with both uncoated and AlCrNi-coated aluminum electrodes. Their research revealed current as the most important input element in the EDM process. Nguyen et al.^21^ used the Taguchi-TOPSIS optimization approach to establish the best process parameters for micro-EDM machining of titanium alloys using a tungsten carbide electrode. Their studies demonstrated that electrode rotation improved machinability, surface quality, and machining accuracy. Dash et al.^22^ evaluated the influence of EDM machining settings on MRR and SR using experimental approaches and the Taguchi method. Their findings indicated that pulse-on time had the greatest influence on MRR, followed by current, pulse-off time, and TiC concentration in the composite. In contrast, for decreasing SR, pulse-off time was the most important element, followed by current, pulse-on time, and TiC concentration. While the experimental MRR values closely matched Taguchi-based predictions, the predicted SR values were lower than the experimental values, indicating that SR is more susceptible to machining factors. Dash et al.^23^ undertook a more in-depth examination into major parameters impacting MRR and SR, with ANOVA findings supporting pulse-on time as the most important element affecting both responses. Their investigation also found fluctuations in MRR and SR when EDM input parameters changed.

The literature study highlights that the application of soft computing approaches for optimizing surface roughness in electrical discharge machining of magnesium/titanium carbide metal matrix composites (range from 0 to 20% TiC in 5% increments) is still inadequately investigated. To get consistent findings about surface roughness, a more extensive experimental dataset and more adjustment of process parameters are required. In the backdrop of this, the present work is directed towards exploring the hybrid optimization technology by application of ANN integrated with the JAYA algorithm for minimization of *SR* in die-sinking EDM of Mg/TiC MMCs considering three machine input process parameters (pulse on time, pulse off time, and current) and weight percentage of TiC. The MMC material was fabricated with varied weight percentages of TiC (0-5-10-15-20%) is also unique and considered as one of the variables in the optimization process which was not reported by previous researchers and this work is being done to close the gap. The novelty of the present work lies in the application of an integrated methodology combining ANN modeling technique with Jaya optimization algorithm for minimization of SR in EDM of MMC (Mg/TiC) and has seldom been attempted by past researchers.

## EDM experimental work on Mg/TiC MMCs

The MMC used in the present work consists of magnesium alloy (AZ91D) as matrix and TiC as reinforcement with varying weight percentage (*WP*) from 0 to 20% in steps of 5%. The composition of workpiece and tool electrode is shown in the Tables 1 and 2 respectively.

**Table 1:** Composition of workpiece.

Sample no	Composition of workpiece	
	Magnesium (%)	TiC (%)
1	100	0
2	95	5
3	90	10
4	85	15
5	80	20

**Table 2:** Composition of workpiece.

Composition of tool electrode	
Copper (%)	Cadmium (%)
99	1

Before casting the composites, the magnesium alloy (AZ91D) is cut into tiny pieces, which are then fed into a graphite crucible that is situated inside a vertical bottom pouring induction furnace. After that, the alloy is heated in an inert argon shield to a temperature of roughly 500–550 °C over the metal’s melting point. The reinforcing element TiC particles, is warmed in a muffle furnace at 1400 °C for two hours in order to avoid any contamination from moisture and other particles. After that, the heated TiC particle is progressively introduced to the liquid matrix. A stirrer is used to ensure that the matrix and reinforcement are properly mixed at high temperatures. The melt is kept at 650 °C, which is superheated, and then it is put into the mold at the same temperature. Accordingly, Mg/TiC MMC specimens with 0%, 5%, 10%, 15%, and 20% of TiC by weight are synthesized through the stir casting method in the previous study by Dash et al.^24^.

**Fig. 1:**
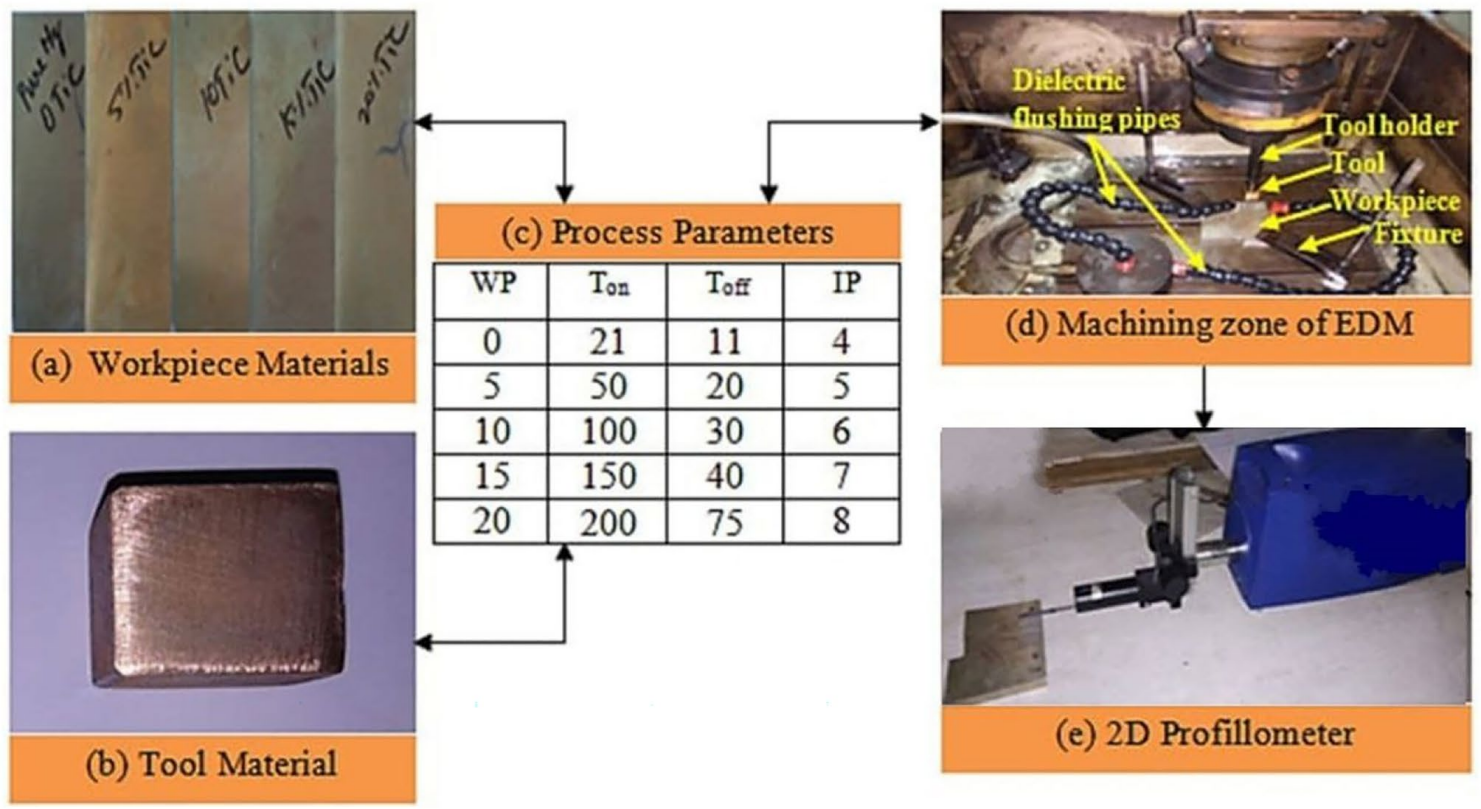
(**a**) Workpiece, (**b**) Tool, (**c**) Process parameters, (**d**) Machining zone, (**e**) 2D SR Profilometer.

**Fig. 2:**
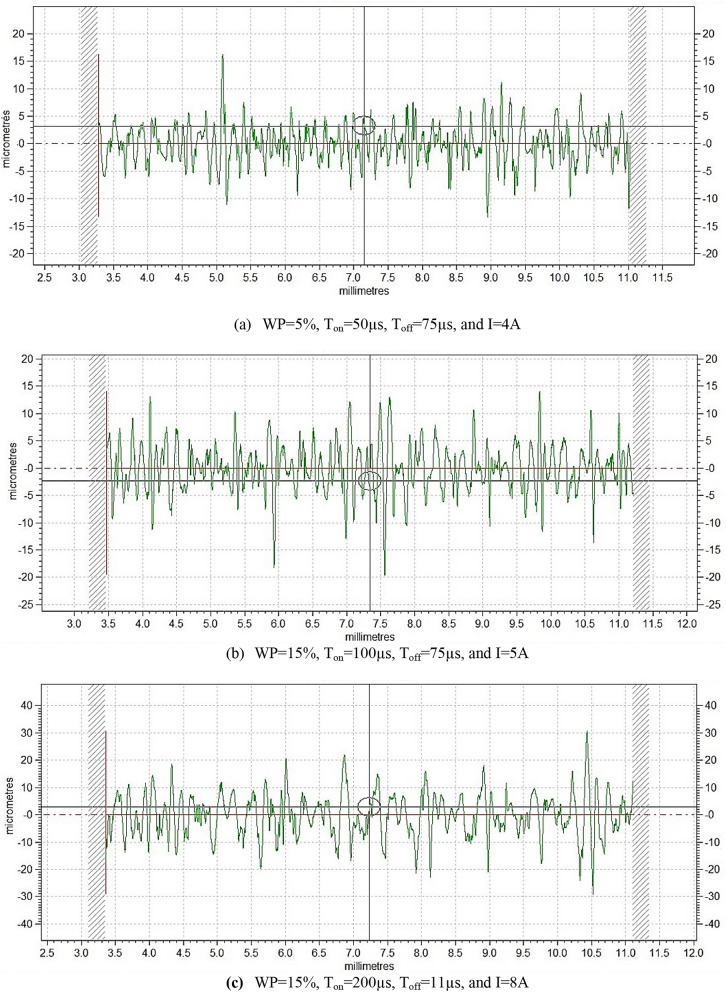
Surface roughness plot (**a**) Experiment No. 10, (**b**) Experiment No. 15, (**c**) Experiment No. 21.

Figure 1a illustrates the work materials and the electrode material used in the present work is pure copper (99%) and is depicted in Fig. 1b. The working dimension of the electrode was 18.72 $\times$ 18.72 mm^2^. A spark EDM of type SPARKONIX MOS 25A with a servo head was used for experimentation whereas commercial kerosene was used as a dielectric fluid. Apart from the *WP* of reinforcement, pulse on time (*T*_*on*_) in μs, pulse off time (*T*_*off*_) in μs, and current (*I*) in Amp are considered EDM process parameters with a range as shown in Fig. 1c. The process parameters range was chosen on the basis of machine tool (SPARKONIX MOS 25A) limitations and literature reviewed^4,10^. The selection of a suitable design of experiments is necessary to minimize experimental run. In this work, Taguchi L_25_ orthogonal array was employed to conduct EDM experiments. Table 3 delineates the diverse combinations of input process parameters developed in accordance with the L25 experimental design, utilizing Minitab statistical software. Figure 1d depicts the EDM working zone. In the machining process, the Mg/TiC metal matrix composite (MMC) workpiece is firmly secured in a fixture, while the copper electrode functioning as the tool, is retained by the tool holder. Before cutting, the electrode is manually aligned with the workpiece to maintain an automated spark gap of 0.02 to 0.05 mm. The machining process occurs in a dielectric fluid, completely immersing the working area. Process parameters are established in accordance with the L25 experimental design, and during the pulse-on phase, electrical discharges provide thermal energy that melted the workpiece material. A pressured side-flushing system, functioning at 15 kg/cm^2^, is employed during the pulse-off phase to efficiently remove eroded material from the discharge area. This cycle is perpetually repeated until the electrode’s shape is precisely imprinted onto the workpiece surface. Subsequent to each trial run, the machined block is removed and dried (Fig. 2).

**Table 3:** Experimental plan used and measured surface roughness values.

Exp. No	Input parameters				Output response
	Weight percentage (*WP*) (%)	Current (I) (Amp)	Pulse on time *(T*_*on*_) (μs)	Pulse off time (*T*_*off*_) (μs)	Surface roughness (*R*_*a*_) (μm)
1	0	4	21	11	3.09
2	5	5	21	20	2.48
3	10	6	21	30	3.53
4	15	7	21	40	4.14
5	20	8	21	75	3.38
6	10	5	50	11	2.80
7	15	6	50	20	3.06
8	20	7	50	30	3.19
9	0	8	50	40	3.40
10	5	4	50	75	2.69
11	20	6	100	11	3.81
12	0	7	100	20	3.53
13	5	8	100	30	3.73
14	10	4	100	40	2.61
15	15	5	100	75	3.33
16	5	7	150	11	3.20
17	10	8	150	20	5.98
18	15	4	150	30	4.29
19	20	5	150	40	4.32
20	0	6	150	75	4.85
21	15	8	200	11	6.12
22	20	4	200	20	5.02
23	0	5	200	30	6.20
24	5	6	200	40	6.56
25	10	7	200	75	6.57

Surface roughness is an important quality characteristic used to describe surface quality of the final product. It is widely used as a vital criterion to distinguish and select the finished components. In the present work, the center-line average (CLA) value of SR is considered as given in Eq. (1).

1 $${R}_{a}=\frac{1}{{L}_{s}}{\int }_{0}^{{L}_{s}}\left|{Y}_{SR}\left(x\right)\right|dx$$

The centerline average (CLA) value of surface roughness (SR) is determined using the relationship between the sampling length (Ls) and the roughness profile ordinate (YSR). A two-dimensional (2D) profilometer (Tylor Hobson, Talysurf 50) was employed to assess the CLA value of SR over a machining span of 8 mm, as illustrated in Fig. 1e. Measurements were conducted at three distinct locations on the machined surface, and the average value was considered the final SR measurement. The experimentally obtained SR values are presented in Table 1. Among the tested parameter configurations, the experimental run designated as Expt. No. 2 (T_on_ = 21 µs, WP = 5%, T_off_ = 20 µs, I = 5A) resulted in the lowest surface roughness, with Ra measured at 2.48 µm. Conversely, the maximum roughness value of Ra = 6.57 µm was observed under the cutting conditions specified in Expt. No. 25 (T_on_ = 200 µs, WP = 10%, T_off_ = 75 µs, I = 7A).

**Fig. 3:**
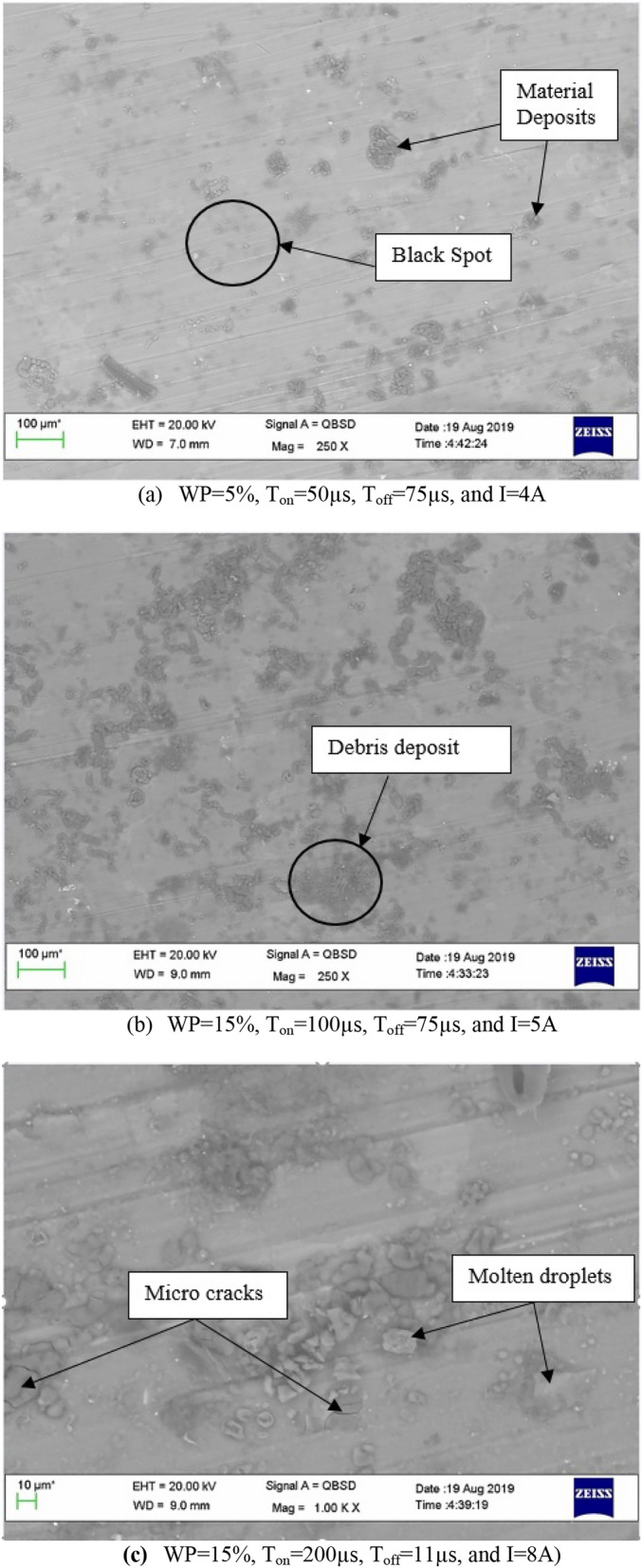
SEM micrograph for (**a**) Experiment No. 10, (**b**) Experiment No. 15, (**c**) Experiment No. 21.

To analyze the surface properties, SEM micrographs of the EDM surfaces are captured. The SEM micrograph of the EDMed surface for experiments nos. 10, 15, and 21 is displayed in Fig. 3. The discharged energy during the EDM process causes extremely high temperatures at the spark point, which melts and vaporizes a little portion of the sample. Figure 3a shows SEM micrographs indicate the existence of minute black spots on the machined surface, suggesting that residual material persists post-machining and is inadequately removed by the dielectric fluid. Pulse on time, pulse off time, and peak current are important electrical characteristics that changed the surface topography. The ongoing accumulation of debris is directly attributable to prolonged pulse-on time and input current, resulting in recurrent spark contacts with the workpiece and which results in accumulation of more and more debris particle on the machined surface as shown in the Fig. 3b. As the pulse-on period and input current further extends, a greater amount of debris collects on the machined surface due to extended spark contact. In the pulse-off phase, insufficient dielectric pressure may impede efficient debris removal, resulting in residual hot particles clinging to the workpiece and solidifying as molten droplets, and micro-cracks as shown in the Fig. 3c.

The white layer is considered to be the molten material that re-solidifies on the workpiece surface during the pulse on time but is not flushed out of the EDMed zone. The two most important factors influencing the white layer are the peak current and the pulse-on time^25^. For experiments No. 10, 15, and 21 as shown in Table 3 it can be observed that the current and pulse on time rises which in turn is responsible for the production of additional white layers as observed in the SEM micrograph (Fig. 3). Consequently, the surface integrity declines as the white layer rises.

## Modeling of surface roughness

### Artificial neural network

ANN is a class of soft computing techniques used in diverse fields for data classification, pattern identification, modeling, etc. Inspired by biological neurons of the human brain, ANNs can acquire knowledge from training/experience, store, and use. In manufacturing fields, ANN is used to predict performance measures by modeling process variables and responses. ANN is a popular soft modeling tool that can effectively describe linear and non–linear relationships between process variables and responses which can be trained by experimental data. ANN is a data-intensive modeling tool requiring huge data sets to model the system.

This study investigates a model based on ANN for predicting SR in the EDM of MMC. An ordinary artificial neural network framework consists of three fundamental layers: the input layer, the hidden layer, and the output layer. The input layer enables data entry into the network for processing, while the hidden layer functions as the computational nucleus, where data transformation takes place prior to transmission to the output layer. The efficacy of the ANN model is profoundly affected by the choice of the transfer function and the quantity of neurons in the hidden layer. The output layer produces the ultimate predictions derived from the processed data. This study developed an ANN model to predict the SR of Mg-TiC MMC during the EDM process, taking into account four machining parameters: workpiece (WP), pulse-on time (Ton), pulse-off time (Toff), and discharge current (I). A feed-forward NN with one hidden layer has been employed for this purpose.

### ANN model development

**Fig. 4:**
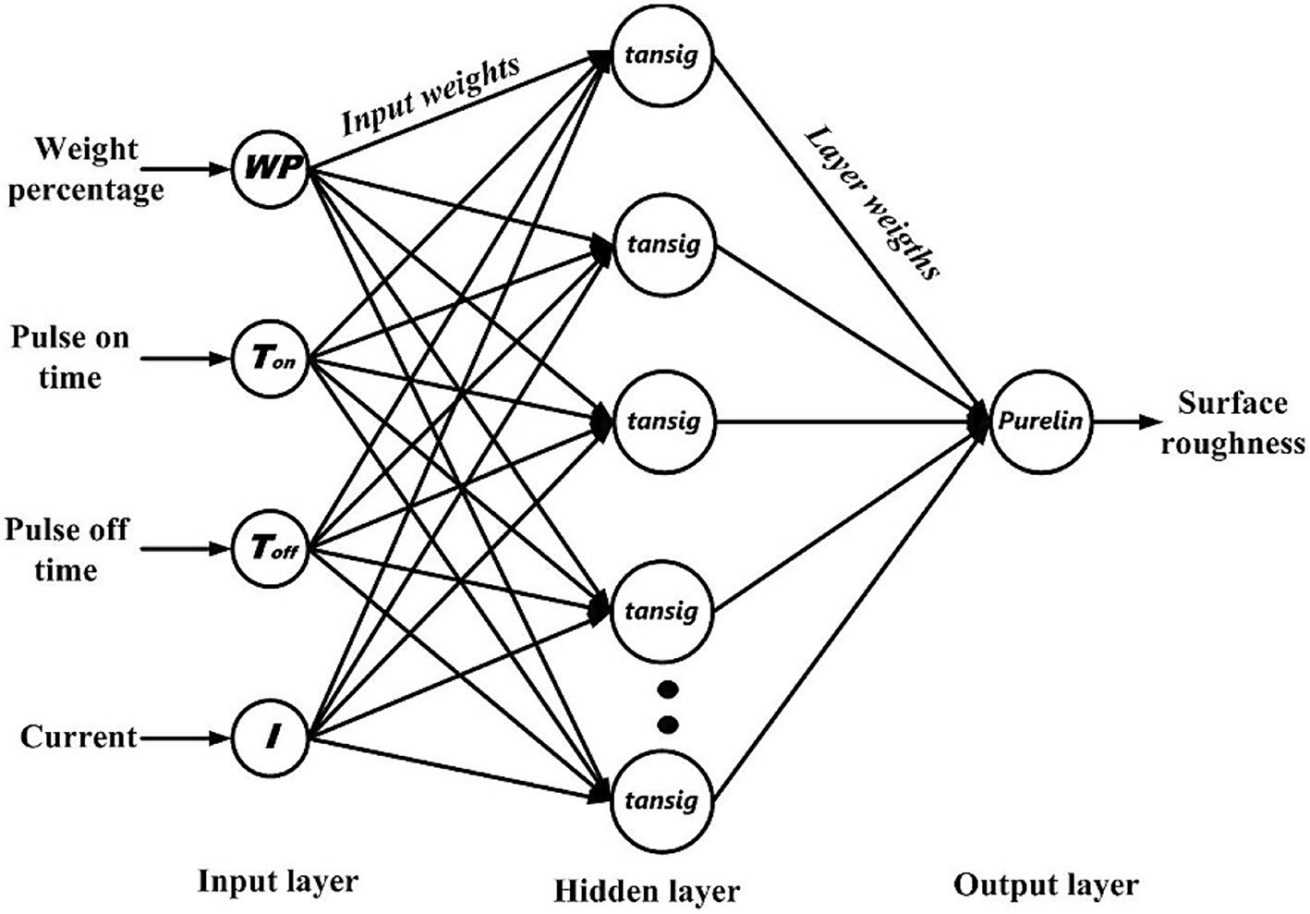
Schematic diagram of ANN architecture used.

In the development of the ANN prediction model, training the network is an important step followed by model validation. It requires a large amount of data for NN development which involves two phases viz., training of NN and testing the trained network. The selection of datasets for NN training and testing is a challenging issue (Kecman et al.^26^). Generally, NN is trained with a set of data sets known as training data and subsequently tested with fresh set of data. Error on training and testing data set is calculated individually and effective error is determined as a difference between average of training error and testing error. NN with the least effective error is considered as optimal network architecture and its predictive performance is tested with the validation data set (Abburi et al.^27^ and Kapli et al.^28^). In this work, data was randomly divided in a ratio of 80:20. Figure 4 shows the ANN architecture used in the present work. It consists of an input layer with four neurons equal to four process variables and an output layer with one neuron representing surface roughness as a performance measure being modeled. Depending on the complexity of the problem being modeled multiple hidden layers can be considered. In this work, NN with one hidden layer is considered. The number of neurons in the hidden layer varied from 5 to 20 with *“log sigmoid”* (*logsig*) and *“tan sigmoid”* (*tansig*) transfer functions. The *logsig* and *tansig* transfer functions employed in the present work are expressed as in Eqs. 2 and 3 respectively. Equation 4 represents the mathematical form of the *purelin* transfer function used in the output layer.

2 $$a = \log sig(b) = \frac{1}{{(1 + e^{ - b} )}}$$

3 $$a = {\text{tansig}}(b) = \frac{2}{{(1 + e^{ - 2b} )}} - 1$$

4 $$a = {\text{purelin}}(b) = b$$

where ‘a’ is the output of the transfer function while ‘b’ is the net weighted input to the neuron. All input datasets were normalized to lie between [− 1, 1] using “*mapminmax*” function. Training and testing error at each NN configuration is determined and effective error is calculated. NN configuration with the least effective error is considered as optimal. The selection of proper training functions is also vital to arrive at optimal NN. In this present work, NN is trained with a *trainbr* (Bayesian regulation) training function as it has good generalization capability with small data sets.

Training and validation datasets were randomly partitioned in an 80:20 ratio using the “dividerand” function. Among the 20 datasets utilized for neural network (NN) model construction, 16 were allocated for training, while the remaining 4 were reserved for testing the trained model. With each execution of the NN, a different selection of training and testing datasets was employed. The network’s performance was assessed based on the mean square error (mse), and the maximum iteration limit was set at 25,000. The MATLAB R2014a platform was used to execute the source code, systematically adjusting the number of neurons in the hidden layer between 5 and 20 while applying various weight combinations to minimize the mse, which propagated backward at the conclusion of each iteration. Table 4 presents the training, testing, and associated errors recorded across different NN configurations. Based on the lowest observed effective error of 2.15%, the 4-tansig 9-1 NN configuration was identified as optimal for the present study on EDM of MMC in predicting SR. This optimal NN model consists of four neurons in the input layer, a single hidden layer with nine neurons utilizing the tansig transfer function, and an output layer with one neuron.

**Table 4:** Training and testing error obtained for selected ANN models.

Sl. No	ANN topology	Mean training error	Mean testing error	Effective error (Abs)
1	4-logsig 4–1	15.92	6.06	9.86
2	4-logsig 8–1	18.56	6.16	12.40
3	4-tansig 7–1	14.09	7.52	6.57
4	4-tansig 9–1	5.89	8.04	2.15
5	4-tansig 12–1	13.02	8.15	4.87
6	4-tansig 17–1	14.92	7.23	7.69

**Fig. 5:**
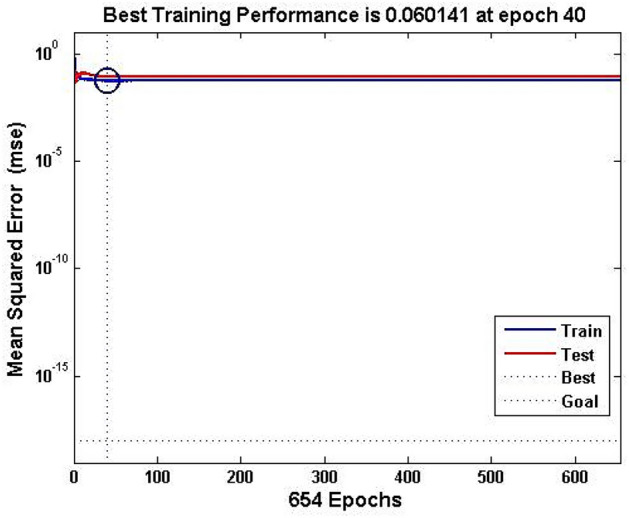
Performance graph during network development.

Figure 5 shows the performance of the ANN model during its model development. MSE is used to evaluate the performance of NN being used. The best MSE was obtained in 40 epochs and it remained constant during the rest of the training epochs. It shows how fast the NN is getting trained and being converged, which can be further used as a prediction model. In ANN training, the term “epoch” refers to “iteration”. In the present work, the maximum iterations (epochs) were set as 25,000. The NN model converged at 654 iterations with the best mse of 0.06 at 40 iterations and the same is depicted in Fig. 3.

**Fig. 6:**
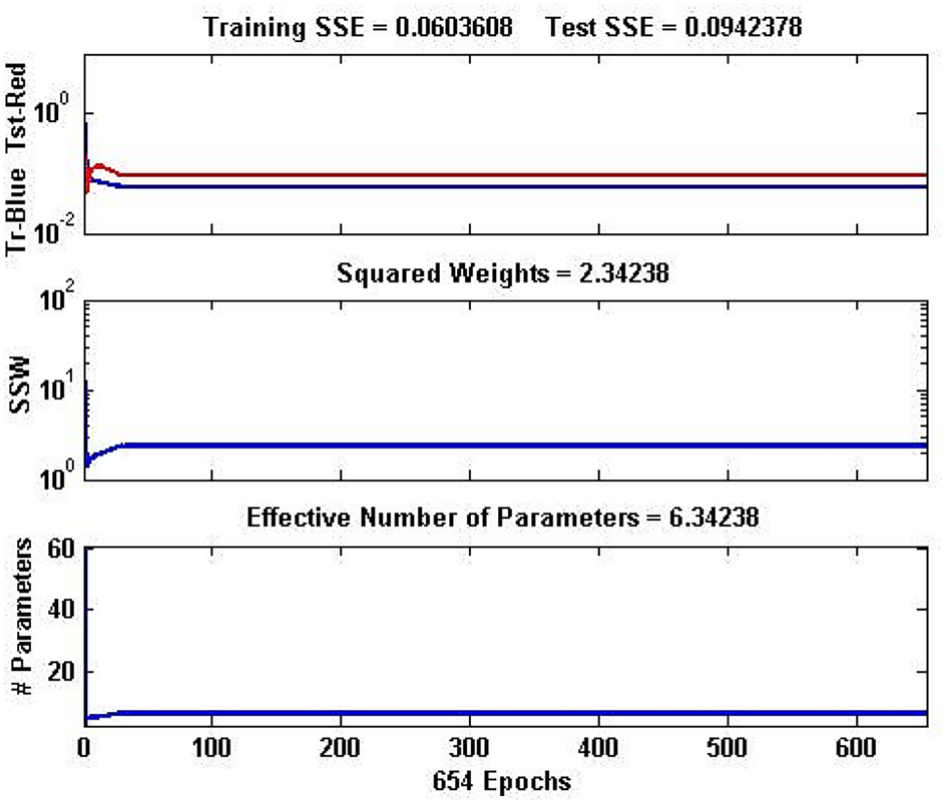
Network parameters of the optimal NN model considered.

Convergence of a NN model can be identified using the network parameters which depend on the training function used. The trainbr training function used in the present work is characterized by the network parameters viz. sum squared error (SSE) on training and testing data sets, sum squared weights (SSW), and an effective number of parameters. The network parameters of the converged optimal NN model are depicted in Fig. 6. It can be seen that SSE for training and testing data sets was obtained as small as 0.06 and 0.09 respectively, in less than 50 iterations and remained unchanged. The constant values of SSW (2.34) and the effective number of parameters (6.34) over 654 epochs imply that NN has converged and can be used as a prediction model.

**Fig. 7:**
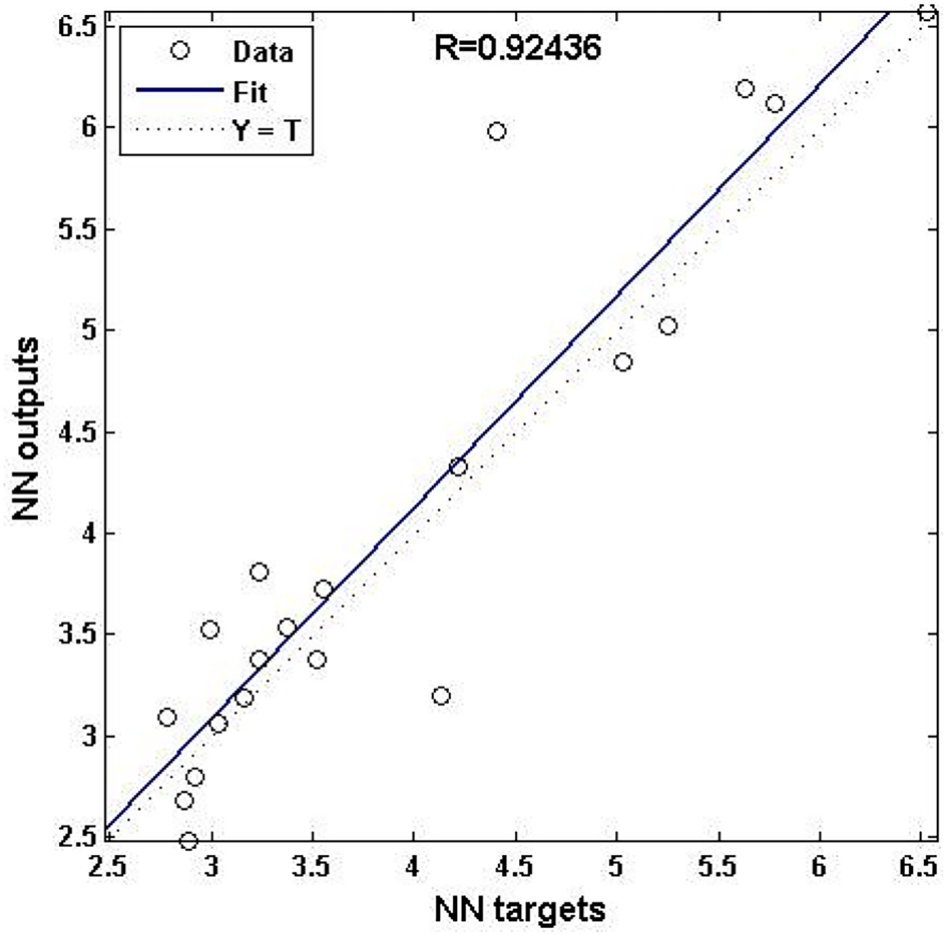
Regression fit of network targets and output values.

The result of NN training for the converged network is presented in Table 5. Percentage errors on training and testing data sets are also given. The average percentage error and mean square error are evaluated using the expression given in Eqs. 5 and 6 respectively.

5 $$APE = \frac{1}{n}\sum\limits_{i = 1}^{n} {\left( {\frac{{|x_{i} - y_{i} |}}{{x_{i} }}} \right)} \times 100$$

6 $$MSE = \frac{1}{n}\sum\limits_{i = 1}^{n} {(x_{i} - y_{i} )^{2} }$$

where *x*_*i*_ is the experimental value for the dataset, *y*_*i*_ is the predicted value and *n* is the number of datasets. The average error on the training and testing data set was obtained as 5.89% and 8.04% respectively. With small *mse* of 0.08 and 0.17 on training and testing data sets and an average percentage error of less than 10%, the developed NN model can be considered to fit experimental datasets accurately in the present study of EDM of MMCs. The correlation coefficient value for the selected optimal NN is obtained as *R* = 0.92 as depicted in Fig. 7. The higher R value achieved suggests that the constructed NN model accurately matches the experimental data in the current study. The generated NN model may be used to estimate SR levels in EDM of MMC for previously unknown datasets in the experimental area. The chosen optimum NN model is tested against five experimental datasets that were not utilized in model construction.

**Table 5:** Result of ANN prediction during model development.

Sl. No	*WP*	*T*_*on*_	*T*_*off*_	*I*	*R*_*a*_ values	Percentage error	
	(%)	(µs)	(µs)	(A)	Expt	ANN	
1	0	21	11	4	3.09	2.78	10.09
2	5	21	20	5	2.48	2.89	16.38
3	10	21	30	6	3.53	3.00	15.06
4	10	50	11	5	2.80	2.92	4.13
5	15	50	20	6	3.06	3.04	0.83
6	15	100	75	5	3.38	3.52	4.14
7	5	50	75	4	2.69	2.87	6.66
8	0	100	20	7	3.53	3.37	4.63
9	20	21	75	8	3.38	3.23	4.35
10	5	150	11	7	4.20	4.13	1.52
11	20	150	40	5	4.32	4.22	2.43
12	0	150	75	6	4.85	5.03	3.66
13	15	200	11	8	6.12	5.77	5.63
14	20	200	20	4	5.02	5.25	4.55
15	0	200	30	5	6.20	5.63	9.20
16	10	200	75	7	6.57	6.52	0.79
17^#^	20	50	30	7	3.19	3.16	1.03
18^#^	20	100	11	6	3.81	3.23	15.23
19^#^	5	100	30	8	3.73	3.56	4.68
20^#^	10	150	20	8	4.98	4.41	11.52

#Testing data sets.

Confidence interval (CI) is the range of values that indicates uncertainty or error of margin around an estimate. Upper and lower limits of CI provide a range of the values within which the population mean is likely to lie. In this work, CI for the training as well as test data sets was calculated and checked whether the mean of the ANN predicted values lie within the range or not. The result of statistical analysis performed to calculate the CI at a 95% confidence level are listed in the Table 6.

**Table 6:** Confidence intervals for training and testing data.

Particulars	Training data	Testing data
Mean	4.08	3.93
Standard error	0.33	0.38
Median	3.53	3.77
Standard deviation	1.32	0.75
Sample variance	1.74	0.57
Range	4.09	1.79
Minimum	2.48	3.19
Maximum	6.57	4.98
Confidence level (95.0%)	0.70	1.20
Upper limits of CI	4.78	5.13
Lower limits of CI	3.37	2.73

The mean of the ANN predicted values for training data sets is obtained as 4.01 and lies within the limits of CI at a 95% confidence level i.e., $3.37\le 4.01\le 4.78$. Similarly, the mean of the ANN predicted values for testing data sets is obtained as 3.59 and lies within the limits of CI at a 95% confidence level i.e., $2.73\le 3.59\le 5.13$. Hence, ANN predicted values of *SR* with an average percentage error of 5.89% (training) and 8.04% (testing) during model development are found statistically acceptable.

## ANN-JAYA integrated optimization methodology

In this section, an integrated optimization methodology combining ANN and Jaya algorithm is proposed for the minimization of *SR* in EDM of MMC. ANN is used as a modeling tool while optimization is carried out using the Jaya algorithm. ANN model predicts *SR* values for MMC at given EDM process variables while process variables are optimized to yield minimum *SR* value using the Jaya optimization technique in a four-dimensional search space.

### JAYA algorithm

**Fig. 8:**
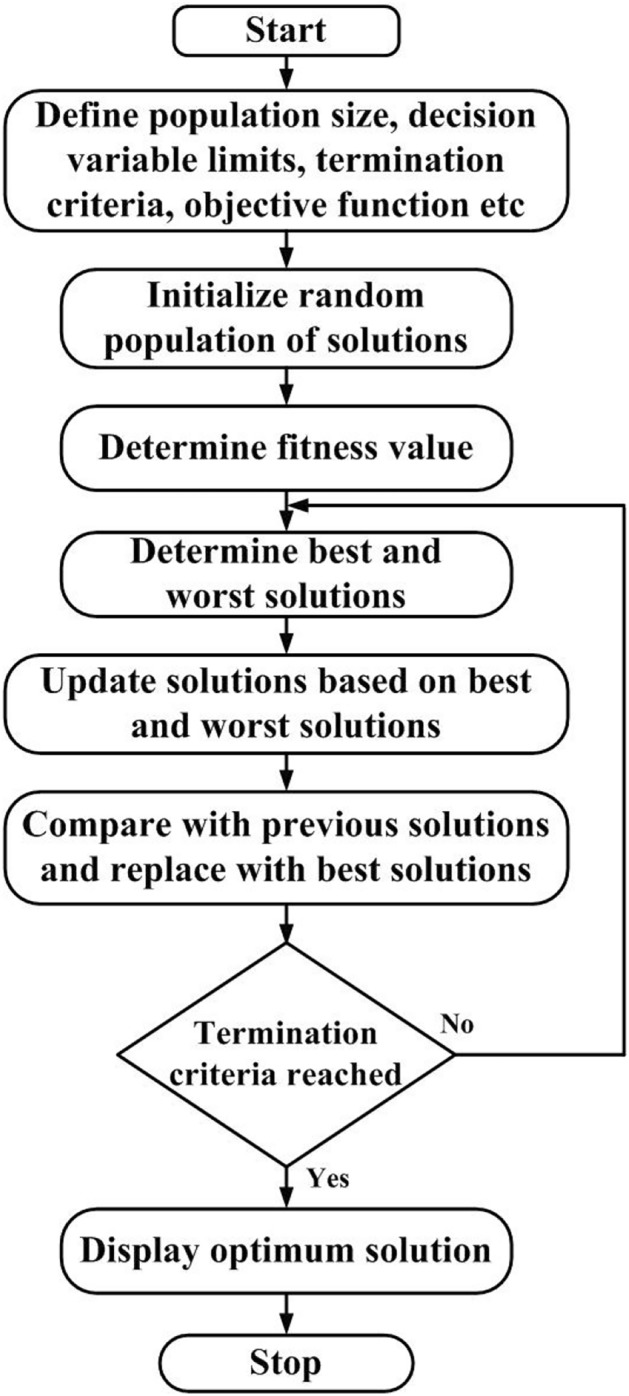
Flow chart of Jaya algorithm.

The Jaya algorithm was first proposed by Rao et al.^29^. Most of the optimization algorithms based on swarm intelligence and evolutionary techniques are probabilistic and require specific control parameters. Genetic algorithm (GA) requires proper selection of mutation and cross-over probability; particle swarm optimization (PSO) requires optimal values related to inertia weight, cognitive and social parameters; artificial bee colony uses control parameters related to different bees (scout, onlooker, and employed bees), etc. The selection of suitable control parameters for a problem under consideration is very crucial for obtaining reliable optimization results. Most of the researchers use default values of control parameters as there are no proper guidelines for selecting suitable values of control parameters specific to a particular problem. Hence, there is a need to develop optimization algorithms free from specific control parameters so that these can be applied to any optimization problem to obtain reliable results. Considering this aspect, Rao et al.^30^ developed a teaching learning-based optimization (TLBO) algorithm that requires no common control parameters except population size and number of iterations. TLBO has gained wide popularity among researchers and is being applied in diverse fields to solve single and multi-objective optimization problems. Inspired by the success and wide acceptability of the TBLO algorithm, Rao et al.^29^ proposed another algorithm called “Jaya” with no specific control parameters. Jaya algorithm is the simplest and easiest to apply. It requires no common parameters and optimization is carried out in a single phase using the characteristic expression given in Eq. 7.

7 $${X}_{new,i}={X}_{i}+{r}_{1}\left({X}_{best,i}-\left|{X}_{i}\right|\right)-{r}_{2}\left({X}_{worst,i}-\left|{X}_{i}\right|\right)$$

where, ${X}_{new,i}$ is the updated new value of the function variable ${X}_{i}$ at ${i}^{th}$ iteration; ${X}_{best,i}$ and ${X}_{worst,i}$ are the best and worst solutions. The flow chart of the Jaya algorithm is depicted in Fig. 8. The solutions are updated by moving toward the best solution as indicated in the first part of the equation while simultaneously moving away from the worst solution as represented by the second part of the expression. The fitness values of the updated solutions are determined compared with the respective fitness values of previous solutions. All the solutions having the best fitness values were stored and used as input for the next iteration. In this way, solutions are updated iteration after iteration until a termination criterion is met.

### Proposed ANN—JAYA integration methodology

**Fig. 9:**
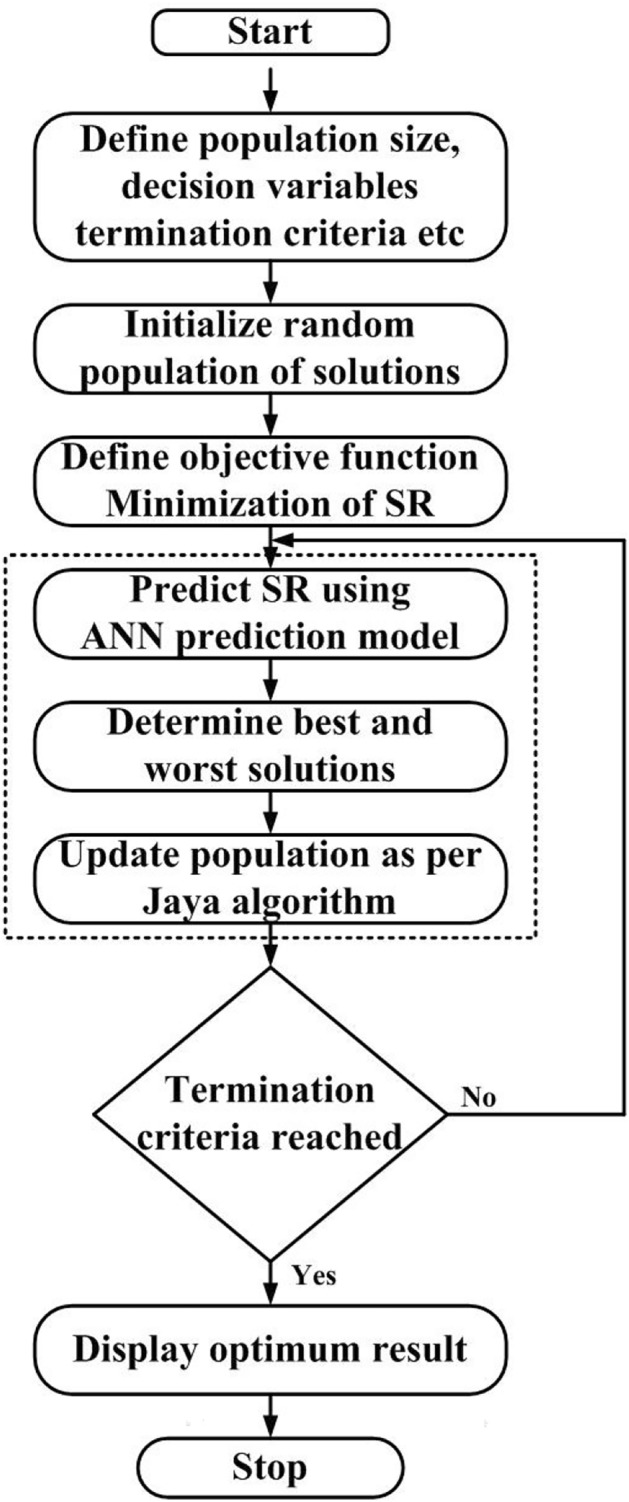
Flow chart of ANN-Jaya integrated optimization methodology.

**Fig. 10:**
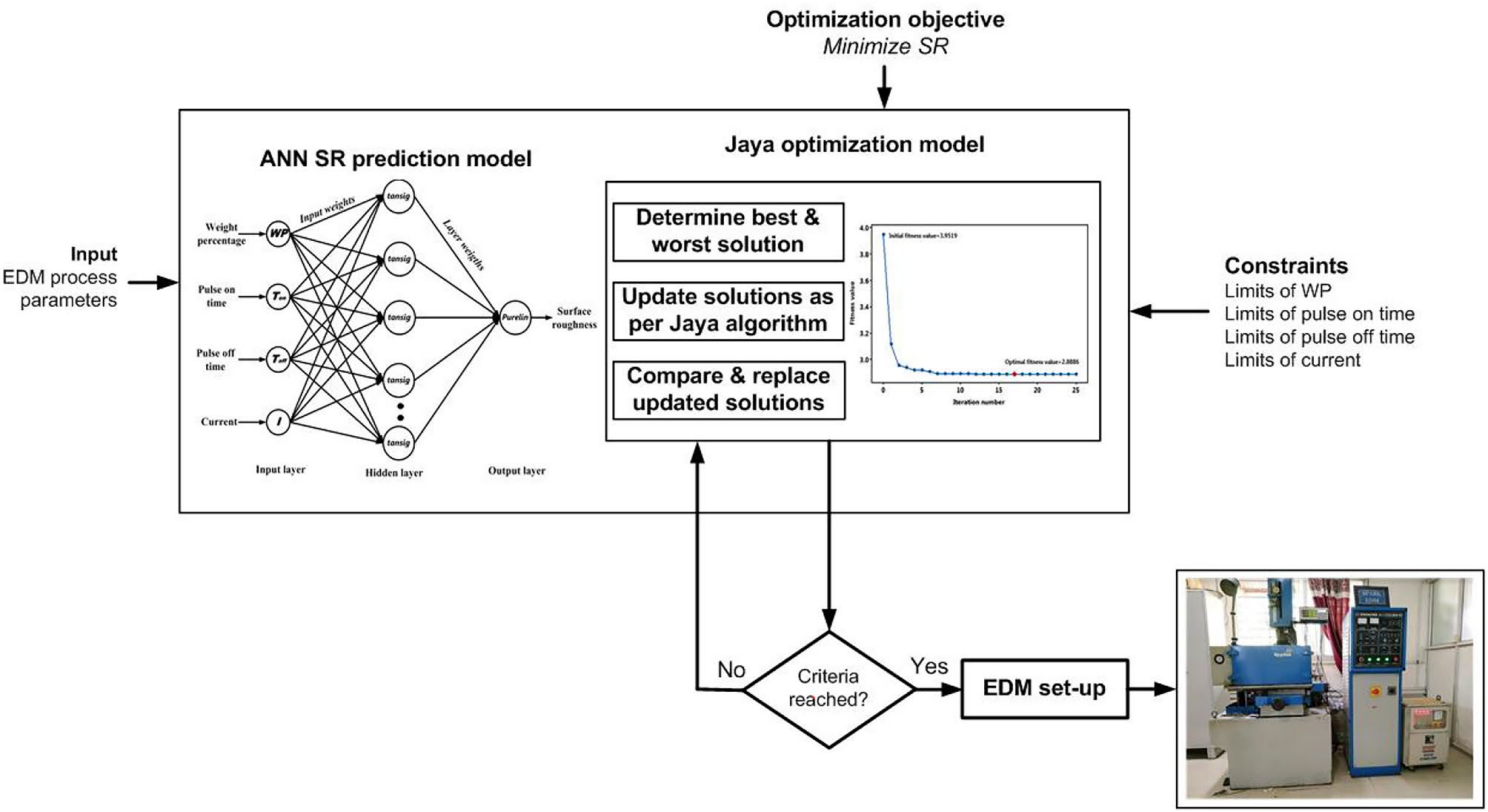
Schematic diagram of the proposed integrated optimization methodology.

Figure 9 illustrates the flow chart of the proposed ANN-JAYA integrated optimization methodology combining ANN as a modeling technique and Jaya as an optimization algorithm for the minimization of *SR* in EDM of MMC. The schematic diagram of the proposed hybrid optimization methodology is depicted in Fig. 10. The motivation for the present work is derived from the published work of the authors Devarasiddappa and Chandrasekaran^31^. The authors have presented a fuzzy logic-PSO integrated optimization approach in the end milling of Al-SiCp MMC in which fuzzy logic was used as a prediction tool while parameters were optimized using the PSO algorithm.

The objective function for minimization of *SR* in EDM of MMC is formulated as given in Eq. (8).

8 $$Minimize SR (WP, {T}_{on,}{T}_{off,}I)$$

Subjected to

9 $${WP}_{min}\le WP\le {WP}_{max}$$

10 $${T}_{on\_min}\le {T}_{on}\le {T}_{on\_max}$$

11 $${T}_{off\_min}\le {T}_{off}\le {T}_{off\_max}$$

12 $${I}_{min}\le I\le {I}_{max}$$

A random population of solutions is generated within the limits of process variables. The ANN prediction model predicts *SR* values for all the solutions of the initial population which is treated as the fitness value of the objective function. The solution matching to the lowest fitness value is regarded the best, while the solution corresponding to the highest fitness value is deemed the worst. Now, all of the original population’s solutions are updated in accordance with Eq. (7). Fitness values i.e., *SR* values of the updated population are determined using the ANN model and are compared with the fitness values of previous solutions. Solutions having minimum fitness values are determined and stored. In this manner, solutions are updated iteration after iteration till a termination condition is fulfilled. The final updated solution having a minimum fitness value is considered an optimal solution.

## Results and discussions

### Machining analysis for SR

In EDM process, the material undergoes a superheating state due to the high temperatures and pressures caused by each sparks during pulse on time in which the material is heated to an above its melting point causes the material to melt and vaporize, which is then carried away by the dielectric fluid. Surface finish of the material is impacted by the energy and intensity of the sparks, which are determined by the pulse on time and current. The increase in pulse on time and input current turns in a rougher surface as observed in the Table 1 and the Fig. 2 shows the surface roughness plot for the experiment no. 10, 15, and 21. It has been observed that, as the values of current and pulse on time increase, the S.R. also increases. Current and pulse-on-time were the most significant parameters affecting S.R. The similar observation was found by Kaigude et al.^32^ irrespective of using dielectric medium as Jatropha oil or EDM oil. The machined impression’s surface roughness becomes coarser as the discharge current (I) and pulse time ratio (PR) increase^33^. SEM analysis was used in this work to identify the EDMed surface following machining, and it was found that the debris, molten droplets, and micro-cracks that were deposited on the EDM surface. The reason is that during the pulse-off time the dielectric fluid is used to remove debris molten particle but unable to remove all the particles from the machining zone.

### Validation of ANN model

The predictive performance of the developed ANN model is evaluated by conducting confirmation experiments. Network output on confirmation datasets is determined and compared with experimental datasets. The result of the validation test is presented in Table 7. The minimum error during model validation was obtained as 1.39% while the maximum error was recorded as 13.85%. With the average percentage error (6.95%) during the confirmation test recorded at less than 10%, the developed ANN surface roughness prediction model in EDM of MMCs can be considered satisfactory.

**Table 7:** Validation of the result of the ANN model.

Sl. No	Process parameters				Surface roughness (µm)		Percentage error
	*WP* (%)	*T*_*on*_ (µs)	*T*_*off*_ (µs)	*I* (A)	Expt	ANN	
1	20	200	20	4	5.02	5.09	1.39
2	0	50	40	8	3.40	3.19	6.18
3	10	100	40	4	3.61	3.11	13.85
4	15	150	30	4	4.29	3.86	10.02
5	5	200	40	6	6.60	6.38	3.33

Model adequacy (MA) is calculated using Eq. (13) as a mean of prediction accuracy on confirmation datasets (Chandrasekaran et al.^34^). With a higher model accuracy of 93.05%, it can be inferred that the developed NN model in the present study is highly accurate.

13 $$MA = \frac{1}{k}\sum\limits_{j = 1}^{k} {\left( {1 - \frac{{\left| {x_{j} - y_{j} } \right|}}{{x_{j} }}} \right)} \times 100$$

### Implementation of the integrated optimization methodology

The proposed ANN-Jaya integrated optimization methodology is applied to obtain global minima of *SR* in the EDM of MMC. EDM experiments were performed by varying, $WP$, ${T}_{on}$, ${T}_{off}$ and $I$ at five different levels as per Taguchi’s L_25_ experimental plan. Implementation of the proposed optimization methodology in the present work consists of the following steps.

*Step 1* Generate a random population of solutions.

A population of random solutions is generated using population size, number of process parameters, upper and lower limits of process variables, etc. The generated random solutions were considered as the initial population. In the present investigation, EDM process parameters were studied $as WP$, ${T}_{on}$, ${T}_{off}$ and $I$. Hence the number of process parameters was taken as 4. The population size was considered as 30. Higher and lower bounds of process parameters were taken as $0\le WP\le 20$, $21\le {T}_{on}\le 200$, $11\le {T}_{off}\le 75$, $4\le I\le 8$ based on EDM set-up configuration. The randomly generated initial population is presented in Table 8. The upper limit of iterations taken as 100 was set as a termination criterion. Accuracy in the fitness value of objective function was considered as another termination criterion.

**Table 8:** Initial population of solutions.

Sr. No	*WP* (%)	*T*_*on*_ (µs)	*T*_*off*_ (µs)	*I* (A)	Fitness value (µm)	Best/worst
1	14.34	135.63	46.91	6.05	5.48	
2	15.29	196.76	66.96	6.96	5.48	
3	1.27	121.25	74.71	5.24	5.80	
4	**12.12**	**63.41**	**66.75**	**7.04**	**5.87**	**Worst**
5	6.46	184.65	12.09	8.00	5.18	
6	17.50	176.11	20.74	7.20	5.23	
7	10.78	145.77	41.21	4.15	5.43	
8	8.63	196.14	47.87	5.27	5.39	
9	**18.79**	**41.20**	**66.68**	**5.40**	**3.95**	**Best**
10	13.88	173.55	23.64	4.92	5.26	
11	0.27	147.66	29.89	4.18	5.38	
12	10.44	126.20	43.85	7.64	5.47	
13	3.94	98.72	42.87	6.88	5.60	
14	14.77	184.17	61.18	5.60	5.48	
15	10.00	142.29	68.48	4.61	5.63	
16	3.16	55.12	74.42	7.02	4.77	
17	2.53	39.77	17.63	5.26	5.62	
18	6.03	171.07	66.74	5.33	5.54	
19	7.08	119.70	56.80	4.62	5.62	
20	13.52	56.01	62.91	6.69	5.55	

Significant values are in bold.

*Step 2* Determine the best and worst fitness solution.

The fitness value of the initial population is determined using the developed ANN prediction model. The solution corresponding to the best and worst fitness value is determined. The predicted fitness value of the initial population is presented in Table 6. The best and worst solution was obtained as $WP=18.79$, ${T}_{on}=41.20$, ${T}_{off}=66.68$, $I=5.40$ and $WP=12.12$, ${T}_{on}=63.41$, ${T}_{off}=66.75$, $I=7.04$ corresponding to fitness values of 3.95 and 5.87 respectively.

*Step 3* Update solutions.

This is an important step in the proposed integrated optimization methodology. Here, solutions are updated based on the best and worst solutions as per Eq. (6). The updated solutions and their fitness values predicted using the ANN model are given in Table 7.

*Step 4* Determine the fitness values of updated solutions and compare.

The fitness values of updated solutions obtained in previous step 3 are compared with the fitness values of previous solutions. Solutions of the updated population having minimum *SR* value were retained while other solutions were replaced with previous solutions. This completes the iteration.

*Step 5* Check termination criteria.

The optimization procedure is terminated if one of the termination criteria is satisfied. Otherwise, the iterative procedure is continued by repeating steps 2 to step 4.

It can be seen from Table 9, that the fitness value (FV) of updated solutions after 1^st^ iteration has improved as compared to its previous solutions. The best FV in the updated population is obtained as 3.12 at $WP=20.00$, ${T}_{on}=2.35$, ${T}_{off}=66.66$, $I=4.19$ which is an improvement over the best FV of the initial population. The FV of the modified population’s worst solution has likewise improved to 5.71 from 5.87 in the initial population. The population of solutions is updated in this fashion iteratively until a termination requirement is met.

**Table 9:** Updated solutions (1st iteration).

Sl. No	*WP* (%)	*T*_*on*_ (µs)	*T*_*off*_ (µs)	*I* (A)	Fitness value (µm)	Best/worst
1	15.43	137.29	46.88	5.41	5.48	
2	20.00	197.63	67.02	6.77	5.48	
3	**5.76**	**134.13**	**75.00**	**5.00**	**5.71**	**Worst**
4	13.93	61.08	66.69	5.65	5.70	
5	11.84	162.96	11.00	7.59	5.17	
6	17.50	176.11	20.74	7.20	5.23	
7	18.51	200.00	37.25	4.00	5.33	
8	8.63	196.14	47.87	5.27	5.39	
9	**20.00**	**25.35**	**66.66**	**4.19**	**3.12**	**Best**
10	13.88	173.55	23.64	4.92	5.26	
11	0.27	147.66	29.89	4.18	5.38	
12	10.44	126.20	43.85	7.64	5.47	
13	3.94	98.72	42.87	6.88	5.60	
14	14.77	184.17	61.18	5.60	5.48	
15	10.00	142.29	68.48	4.61	5.63	
16	10.13	46.55	73.65	6.39	4.02	
17	0.00	34.40	11.00	4.45	5.57	
18	6.11	189.31	66.73	4.00	5.51	
19	7.08	119.70	56.80	4.62	5.62	
20	17.23	47.64	62.60	5.40	4.78	

Significant values are in bold.

Table 10 shows updated solutions and their FV at the end of the 17th iteration. The best and worst FV of the objective function was obtained as 2.89 and 2.95. The obtained best FV of 2.8886 remained unchanged in the successive iterations thereby satisfying one of the termination criteria. Hence, the optimal FV i.e., minimum *SR* obtained in the present investigation in EDM of MMC using ANN-Jaya integrated optimization methodology is 2.89 µm at $WP=8.05\%$, ${T}_{on}=21.00\mu s$, ${T}_{off}=75.00\mu s$, $I=8.00A$.

**Table 10:** Updated solutions (17th iteration).

Sl. No	*WP* (%)	*T*_*on*_ (µs)	*T*_*off*_ (µs)	*I* (A)	Fitness value (µm)	Best/worst
1	8.30	21.00	75.00	8.00	2.89	
2	7.30	21.00	75.00	7.53	2.90	
3	8.50	21.00	75.00	8.00	2.89	
4	7.98	21.00	75.00	8.00	2.89	
5	**9.31**	**22.38**	**67.56**	**7.99**	**2.95**	**Worst**
6	9.04	21.00	75.00	8.00	2.89	
7	8.61	21.00	75.00	8.00	2.89	
8	7.82	21.00	75.00	8.00	2.89	
9	8.35	21.00	75.00	8.00	2.89	
10	8.79	21.00	75.00	8.00	2.89	
11	7.04	21.00	75.00	8.00	2.91	
12	8.30	21.00	75.00	8.00	2.89	
13	8.71	21.00	75.00	8.00	2.89	
14	6.93	21.00	75.00	8.00	2.91	
15	8.88	21.00	75.00	8.00	2.89	
16	8.79	21.00	75.00	8.00	2.89	
17	8.83	21.00	75.00	8.00	2.89	
18	**8.05**	**21.00**	**75.00**	**8.00**	**2.89**	**Best**
19	8.45	21.00	75.00	8.00	2.89	
20	8.25	21.00	75.00	8.00	2.89	

Significant values are in bold.

**Fig. 11:**
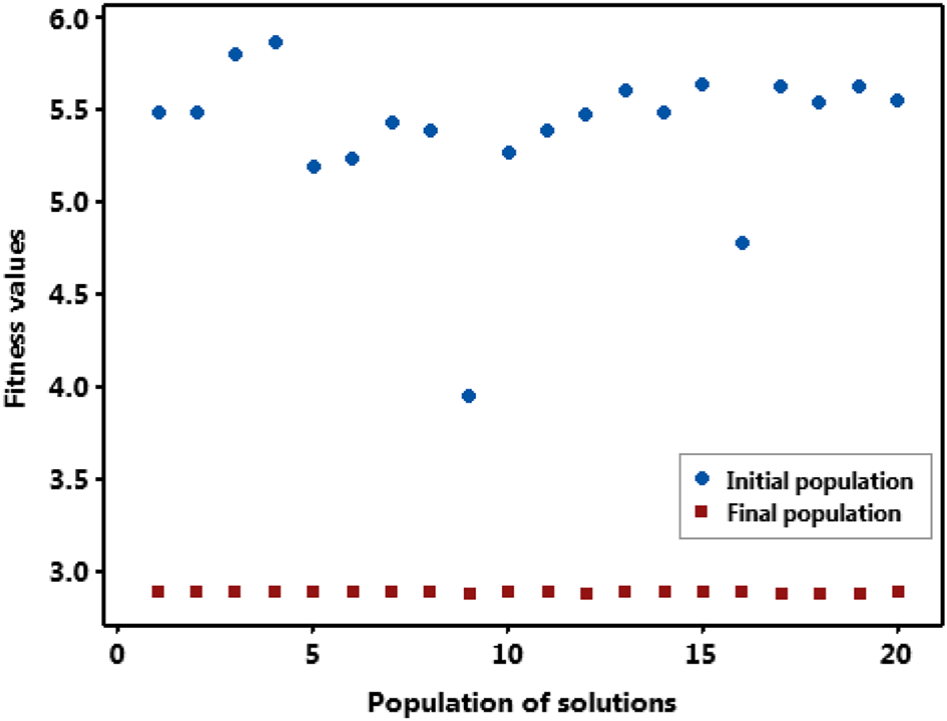
Comparison of fitness initial and final population.

**Fig. 12:**
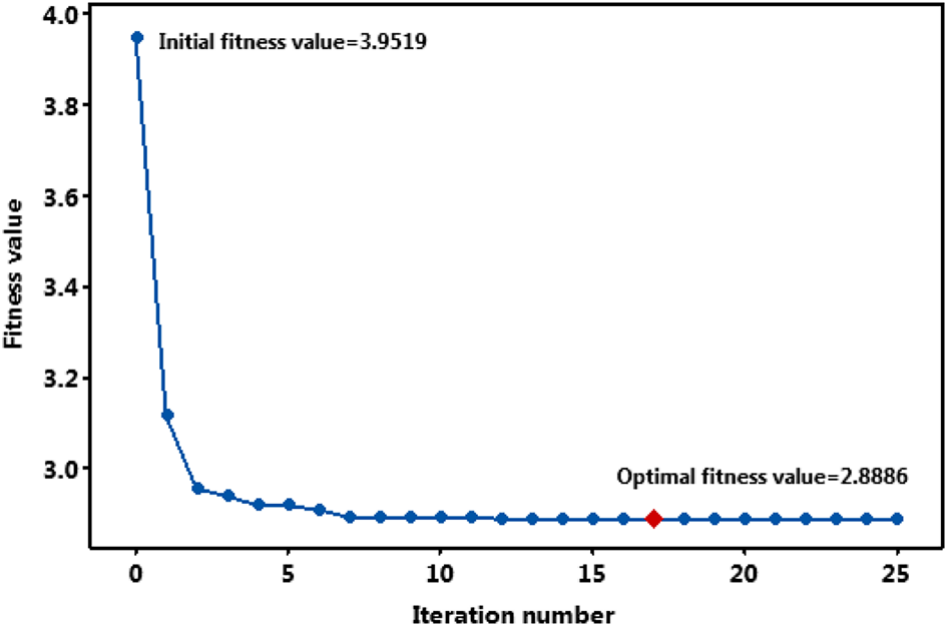
Convergence plot of fitness values.

Table 11 shows the updated population at the end of the 25th iteration. The majority of the solutions converged on the optimal value of 2.89 µm as seen from the tabulated data. Figure 11 shows a graphical representation of the FV of the starting population and the final updated population. The FV of the initial population is widely dispersed, whereas the fitness values of the final population are extremely close to the optimal value of 2.89 µm. Table 12 shows the iteration-by-iteration gain in fitness value during the optimization process, which is graphically depicted in Fig. 12.

**Table 11:** Final updated solutions (25th iteration).

Sl. No	*WP* (%)	*T*_*on*_ (µs)	*T*_*off*_ (µs)	*I* (A)	Fitness value (µm)	Best/worst
1	8.15	21.00	75.00	8.00	2.89	Most of the solutions converged toward the optimal value
2	7.85	21.00	75.00	7.95	2.89	
3	8.13	21.00	75.00	8.00	2.89	
4	8.49	21.00	75.00	8.00	2.89	
5	8.86	21.05	74.69	8.00	2.89	
6	8.34	21.00	75.00	8.00	2.89	
7	8.16	21.00	75.00	8.00	2.89	
8	8.10	21.00	75.00	8.00	2.89	
9	8.06	21.00	75.00	8.00	2.89	
10	8.09	21.00	75.00	8.00	2.89	
11	8.28	21.00	75.00	8.00	2.89	
12	8.07	21.00	75.00	8.00	2.89	
13	8.21	21.00	75.00	8.00	2.89	
14	8.17	21.00	75.00	8.00	2.89	
15	8.15	21.00	75.00	8.00	2.89	
16	8.14	21.00	75.00	8.00	2.89	
17	8.02	21.00	75.00	8.00	2.89	
18	8.03	21.00	75.00	8.00	2.89	
19	8.05	21.00	75.0000	8.00	2.89	
20	8.25	21.00	75.00	8.00	2.89

**Table 12:** Result of consistency of test.

Run/Iteration	1	2	3	4	5	6	7	8	9	10
1	2.92	3.61	3.04	3.77	3.24	3.17	2.90	2.93	3.21	5.17
2	2.90	2.94	3.04	3.18	3.13	3.16	2.89	2.93	3.01	4.68
3	2.89	2.91	2.90	2.89	3.11	3.04	2.89	2.92	2.96	3.04
4	2.89	2.90	2.89	2.89	3.07	2.96	2.89	2.92	2.93	3.04
5	2.89	2.89	2.89	2.89	3.01	2.90	2.89	2.91	2.92	2.93
6	2.89	2.89	2.89	2.89	2.99	2.89	2.89	2.91	2.90	2.93
7	2.89	2.89	2.89	2.89	2.94	2.89	2.89	2.91	2.89	2.93
8	2.89	2.89	2.89	**2.89**	2.94	2.89	2.89	2.91	2.89	2.93
9	2.89	2.89	2.89	2.89	2.94	2.89	2.89	2.91	**2.89**	2.93
10	2.89	2.89	2.89	2.89	2.92	2.89	2.89	2.91	2.89	2.90
11	2.89	2.89	2.89	2.89	2.92	2.89	**2.89**	2.91	2.89	2.89
12	2.89	**2.89**	**2.89**	2.89	2.89	**2.89**	2.89	2.91	2.89	2.89
13	2.89	2.89	2.89	2.89	2.89	2.89	2.89	2.90	2.89	2.89
14	2.89	2.89	2.89	2.89	2.89	2.89	2.89	2.90	2.89	2.89
15	2.89	2.89	2.89	2.89	2.89	2.89	2.89	2.89	2.89	2.89
16	2.89	2.89	2.89	2.89	2.89	2.89	2.89	2.89	2.89	2.89
17	2.89	2.89	2.89	2.89	**2.89**	2.89	2.89	2.89	2.89	2.89
18	2.89	2.89	2.89	2.89	2.89	2.89	2.89	2.89	2.89	2.89
19	2.89	2.89	2.89	2.89	2.89	2.89	2.89	2.89	2.89	2.89
20	**2.89**	2.89	2.89	2.89	2.89	2.89	2.89	2.89	2.89	2.89
21	2.89	2.89	2.89	2.89	2.89	2.89	2.89	**2.89**	2.89	**2.89**
22	2.89	2.89	2.89	2.89	2.89	2.89	2.89	2.89	2.89	2.89
23	2.89	2.89	2.89	2.89	2.89	2.89	2.89	2.89	2.89	2.89
24	2.89	2.89	2.89	2.89	2.89	2.89	2.89	2.89	2.89	2.89
25	2.89	2.89	2.89	2.89	2.89	2.89	2.89	2.89	2.89	2.89
26	2.89	2.89	2.89	2.89	2.89	2.89	2.89	2.89	2.89	2.89
27	2.89	2.89	2.89	2.89	2.89	2.89	2.89	2.89	2.89	2.89
28	2.89	2.89	2.89	2.89	2.89	2.89	2.89	2.89	2.89	2.89
29	2.89	2.89	2.89	2.89	2.89	2.89	2.89	2.89	2.89	2.89
30	2.89	2.89	2.89	2.89	2.89	2.89	2.89	2.89	2.89	2.89
Optima	2.89	2.89	2.89	2.89	2.89	2.89	2.89	2.89	2.89	2.89
Iteration no	20	12	12	8	17	12	11	20	9	20

Significant values are in bold.

### Accuracy and consistency test of the integrated optimization methodology

**Fig. 13:**
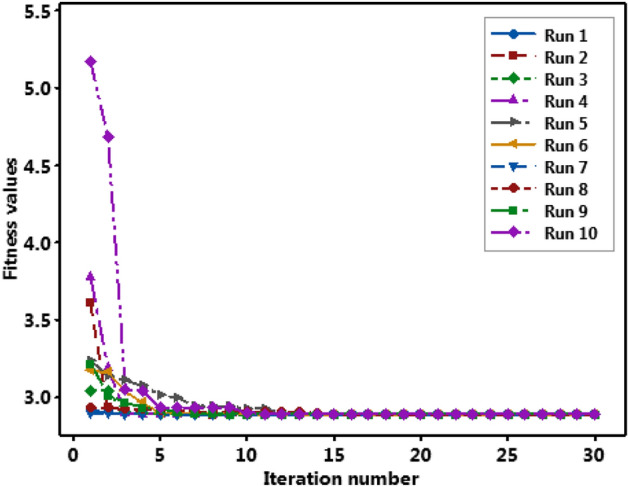
Convergence plot during consistency test.

Application of ANN-Jaya integrated hybrid optimization methodology resulted in a minimum *SR* of 2.89 µm in EDM of MMCs considered in the present study. The optimal value of *SR* was obtained in the 17th iteration itself and remained unchanged during successive iterations. The consistency and accuracy of the optimization methodology were tested by running the algorithm ten times with different randomly generated populations (Devarasiddappa et al.^31^). The obtained optimal value of *SR* and the number of iterations taken to converge were also recorded. The result of the consistency test is presented in Table 10. Figure 13 shows the convergence of fitness values during the consistency test. Mean and standard deviation during the consistency test were obtained as 2.89 and 5.164E-5 respectively. The lower standard deviation indicates that the result of the ANN-Jaya integrated optimization methodology used in this study for minimization of *SR* during EDM of MMC (Mg-TiC) EDM was found to be accurate and consistent. In addition, the proposed optimization approach converged faster, requiring only 8–20 iterations.

In order to minimize SR in die-sinking EDM of Mg/TiC MMCs, the current work intends to investigate hybrid optimization technology by applying ANN in conjunction with the JAYA algorithm. The performance of the suggested ANN-Jaya integrated optimization methodology was found to be extremely accurate and consistent during the consistency test with lower standard deviation, and SR greatly improved as a result of the integrated optimization process.

The surface roughness value estimate model was developed by Ozkavak et al.^35^ utilizing two artificial intelligence techniques, ANN and GEP (gene expression programming). It was determined that the ANN approach was more appropriate than the GEP approach.

## Conclusions

In this research work, minimization of *SR* of machined Mg/TiC MMCs is attempted using a hybrid optimization methodology integrating ANN with the Jaya optimization algorithm. An ANN prediction model was developed to determine *SR* in terms of EDM process parameters viz., pulse on time, pulse off time, and input current, as well as the weight percentage of reinforcement (TiC) in the MMC. The current research work yielded the following significant findings.

- Surface roughness is modeled as a performance measure in EDM of Mg-TiC MMCs with process variables as $WP$, ${T}_{on}$, ${T}_{off}$ and $I$. The parameter setting corresponding to Expt. No. 2 (*T*_*on*_ = 21 µs, *WP* = 5%, *T*_*off*_ = 20 µs, and *I* = 5A) yielded the lowest value of surface roughness as *R*_*a*_ = 2.48 µm. The peak surface roughness of *R*_*a*_ = 6.57 µm was obtained at cutting settings in case of run number 25 (*T*_*on*_ = 200 µs, *WP* = 10%, *T*_*off*_ = 75 µs and *I* = 7A).
- An ANN-based *SR* prediction model in EDM of MMC is attempted. ANN prediction model with *4-tansig9-1* architecture was found optimal in the present work with a 2.15% effective error. The constructed ANN model’s validation result revealed an average percentage error of 6.95% while obtaining a model accuracy of 93.05%.
- A proposed ANN-Jaya integrated optimization methodology is applied to minimization of *SR* in the EDM of MMC. According to the integrated optimization process, *SR* significantly improved from the first testing run. The optimal value of *SR* in EDM of MMC was obtained as 2.89 µm at optimal cutting parameters $WP=8.05\%$, ${T}_{on}=21.00\mu s$, ${T}_{off}=75.00\mu s$, $I=8.00A$.
- The consistency and accuracy of the optimization methodology were tested by running the algorithm ten times with different randomly generated populations. The performance of the proposed ANN-Jaya integrated optimization methodology was found highly accurate and consistent during the consistency test with lower standard deviation of 5.164E-5. The optimization algorithm converged faster and took 8–20 iterations during the consistency test.

## Data Availability

The data is available in the manuscript.

## References

[1] Somashekhar, K. P., Ramachandran, N. & Mathew, J. Optimization of material removal rate in micro-EDM using artificial neural network and genetic algorithms. *Mater. Manuf. Processes* **25**(6), 467–475 (2010).

[2] Lin, Y. C., Chow, H. M., Chen, Y. F. & Liu, J. F. Optimal machining parameters of EDM in gas based on response surface methodology. *Int. J. Mater. Sci. Appl.* **5**(6), 241–247 (2016).

[3] Anitha, J., Das, R. & Pradhan, M. K. Multi-Objective Optimization of Electrical Discharge Machining Processes Using Artificial Neural Network. *Jordan J. Mech. Indust. Eng.* **10**(1), 11–18 (2016).

[4] Markopoulos, A. P., Manolakos, D. E. & Vaxevanidis, N. M. Artificial neural network models for the prediction of surface roughness in electrical discharge machining. *J Intell Manuf* **19**, 283–292 (2008).

[5] Prabhu, S., Uma, M. & Vinayagam, B. K. Electrical discharge machining parameters optimization using response surface methodology and fuzzy logic odeling. *J Braz. Soc. Mech. Sci. Eng.* **36**, 637–352 (2014).

[6] Payal, H., Maheshwari, S. & Bharti, P. S. Process modeling of electric discharge machining of inconel 825 using artificial neural network. *Int. J. Mech. Mech. Eng.* **11**(3), 562–566 (2017).

[7] C Roya, KH Syeda, P Kuppan. Machinablity of Al/10%Sic/2.5%Tib2 Metal Matrix Composite with Powder-Mixed Electrical Discharge Machning. Procedia Technology, 2016, 25:1056–1063.

[8] T. Muthuramalingam, NH Phan. Experimental Investigation of White Layer Formation on Machining Silicon Steel in PMEDM Process. Silicon. 10.1007/s12633-020-00740-7.

[9] Jabbaripour, B., Sadeghi, M. H., Faridvand, S. H. & Shabgard, M. R. Investigating the effects of EDM parameters on surface integrity, MRR and TWR in machining of Ti–6Al–4V. *Mach. Sci. Technol.: An Int. Jo.* **16**(3), 419–444. 10.1080/10910344.2012.698971 (2012).

[10] Natarajan, G., Krishnan, G., Seeniappan, K., Lakshmaiya, N. Influence of heat treated manihot esculenta biosilica on friction stir Welded AA 6065-Al2O3 metal matrix composite and microstructural, mechanical, and fatigue analysis. *Mat Res* [Internet]. **28**, e20240473. 10.1590/1980-5373-MR-2024-0473 (2025).

[11] Torres, A., Puertas, I. & Luis, C. J. Modelling of surface finish, electrode wear and material removal rate in electrical discharge machining of hard-to-machine alloys. *Precis. Eng.* **40**, 33–45 (2015).

[12] Chandramouli, S., Shrinivas, B. U. & Eswaraiah, K. Optimization of electrical discharge machining process parameters using taguchi method. *Int. J. Adv. Mech. Eng.* **4**(4), 425–434 (2014).

[13] Prasanna, P., Sashank, T. V. S. S. P., Manikanta, B. & Aluri, P. Optimizing the process parameters of Electrical Discharge Machining on AA7075 – SiC Alloys. *Mater Today: Proceedings* **4**, 8517–8527 (2017).

[14] Habib, S. S. Study of the parameters in electrical discharge machining through response surface methodology approach. *Appl. Math. Model.* **33**, 4397–4407 (2009).

[15] Sidhu, S. S. & Bains, P. S. Study of the Recast Layer of Particulate Reinforced Metal Matrix Composites machined by EDM. *Mater. Today: Proceedings* **4**, 3243–3251 (2017).

[16] Gangil, M. & Pradhan, M. K. Modeling and optimization of electrical discharge machining process using RSM: A review. *Mater. Today: Proc.* **4**, 1752–1761 (2017).

[17] Heidari, S., Afsari, A. & Ranaei, M. A. Increasing wear resistance of copper electrode in electrical discharge machining by using ultra-fine-grained structure. *Trans Indian Inst Met* **73**, 2901–2910. 10.1007/s12666-020-02091-8 (2020).

[18] Lakshmaiya, N., Nadh, V.S., Kaliappan, S. et al. Enhanced tribological performance of AA6018 aluminium composites reinforced with copper chromate exploring ceramic-based strengthening mechanisms. *J Aust Ceram Soc*10.1007/s41779-025-01215-x (2025).

[19] PH Nguyen, DV. Pham. Single objective optimization of die- sinking electrical discharge machining with low frequency vibration assigned on workpiece by taguchi method, Journal of King Saud University – Engineering Sciences, 10.1016/j.jksues.2019.11.001.

[20] NH Phan, PV Dong, HT Dung, NV Thien, T. Muthuramalingam, S Shirguppikar, NC Tam, N Trong. Multi-object optimization of EDM by Taguchi-DEAR method using AlCrNi coated electrode. Int. J. Adv. Manuf. Technol. 10.1007/s00170-021-07032-3.

[21] Nguyen, P. H. et al. Multi-objective optimization of micro EDM using TOPSIS method with Tungsten carbide electrode. *Sadhana* **47**, 133. 10.1007/s12046-022-01900-8 (2022).

[22] Dash, D., Samanta, S. & Rai, R. N. Flexural, dry sliding wear and machinability (EDM) characteristics of AZ91D/TiC (0, 5, 10, 15, & 20 wt.%) MMCs. *Adv. Mater. Proces. Technol.* **8**(3), 3344–3362 (2022).

[23] Dash, D., Devarajaiah, D., Dash, S. K., Samanta, S. & Rai, R. N. Experimental investigation and machining analysis of Mg/TiC composites during EDM. *Composites Theory Practice* **24**, 1 (2024).

[24] Dash, D., Singh, R., Samanta, S. & Rai, R. N. Influence of TiC on microstructure, mechanical and wear properties of magnesium alloy (AZ91D) matrix composites. *J. Sci. Ind. Res.* **79**, 164–169 (2020).

[25] Ghodsiyeh, D., Akbarzadeh, S., Izman, S. & Moradi, M. Experimental Investigation of Surface Integrity after Wire Electro-Discharge Machining of Ti–6Al–4V. *Sadhana* **44**, 1–15 (2019).

[26] Kecman, K. *Learning and soft computing: support vector machines, neural networks, and fuzzy logic models* (The MIT Press, 2001).

[27] Abburi, N. R. & Dixit, U. S. A knowledge based system for the prediction of surface roughness in turning process. *Robotics Comput. Integrated Manuf.* **22**, 363–372 (2006).

[28] Kapil Surani, Natrayan L, Md Irfanul Haque Siddiqui, Abhinav Kumar, Mohd Asif shah, Intesaaf Ashraf. The evaluation of machining performances and surface roughness of TZM-molybdenum superalloy processed by silicon carbide powder mixed EDM process using RSM and ANOVA. *AIP Advances.* **14**(3), 035141. 10.1063/5.0190922 (2024).

[29] Rao, R. V. Jaya: A simple and new optimization algorithm for solving constrained and unconstrained optimization problems. *Int. J. Ind. Eng. Comput.* **7**, 19–34 (2016).

[30] Rao, R. V., Savsani, V. J. & Vakharia, D. P. Teaching–learning-based optimization: A novel method for constrained mechanical design optimization problems. *Computer Aided Design* **43**, 303–315 (2011).

[31] Devarasiddappa, D. & Chandrasekaran, C. Fuzzy logic-integrated PSO methodology for parameters optimization in end milling of Al/SiCp MMC. *J Braz. Soc. Mech. Sci. Eng.* **41**, 222 (2019).

[32] Kaigude, A. R. et al. Surface roughness prediction of AISI D2 tool steel during powder mixed EDM using supervised machine learning. *Sci. Rep.* **14**, 9683. 10.1038/s41598-024-60543-3 (2024). 38678121 10.1038/s41598-024-60543-3 PMC11055908

[33] Ahmed, N. et al. The potentiality of sinking EDM for micro-impressions on Ti-6Al-4V: keeping the geometrical errors (axial and radial) and other machining measures (tool erosion and work roughness) at minimum. *Sci. Rep.* **9**, 17218. 10.1038/s41598-019-52855-6 (2019). 31748565 10.1038/s41598-019-52855-6 PMC6868188

[34] Chandrasekaran, M. & Devarasiddappa, D. Artificial neural network modelling for surface roughness prediction in cylindrical grinding of Al-SiCp metal matrix composites and ANOVA analysis. *Adv. Prod. Eng. Manag.* **9**(2), 59–70 (2014).

[35] Ozkavak, H. V., Sofu, M. M., Duman, B. & Bacak, S. Estimating surface roughness for different EDM processing parameters on Inconel 718 using GEP and ANN. *CIRP J. Manuf. Sci. Technol.* **33**, 306–314. 10.1016/j.cirpj.2021.04.007 (2021).

